# Health Tract, No. 195

**Published:** 1864-04

**Authors:** 


### HEALTH TRACT, No. 195.

### EMANATIONS.

Philosophers have said that light and heat are ponderable bodies, and that although these have been coming out from the sun for six thousand years, that immense illuminary has not appreciably diminished in size.

The sweetest rose of the beautiful May throws out its delightful fragrance from the first flush of the spring morning until dewy eve, and remains as sweet as ever and quite as large.

The face and air of beauty charmed a thousand hearts yesterday; a thousand more feed upon it to-day, and other thousands of eyes will look upon it to-morrow with a lingering rapture, and the next day it will be not less beautiful than it was a week ago.

Influences go out hourly from the wise and good, and as years roll on these influences gather force, while the wise become wiser, and the good better, hour by hour.

So with business men of integrity, of sterling and tried principles, they throw out an influence from themselves which is a power for good in every community, to restrain the wrong-doer, and awe villainy.

All these are "emanations," influences; material, moral, social; there are also "emanations" malign.

In an autumn morning of the sunny South, or amid the flower-clad prairies of the wide-spreading West, or on the shores of our own Northern lakes and inland seas and crystal flowing streams from among the mountains, as delicious as the still air is, it is more so in the cool of the evening after the sun has gone down from the sky; and yet that balmy atmosphere is so loaded with miasmatic poison that it breeds disease and pestilence and death in a night; it will do the same on successive nights, to one or a million of human beings, without any appreciable diminution in either the amount or malignity of its venom; and so ethereal is it that no alembic of the chemist has ever been able to detect its presence, even to the amount of a single atom.

The very sight of filth and squalor and rags, of a victim of the horrifying small-pox, of the wretch whose whole body is a mass of festering corruption—any of these fill the most transient observer with unutterable disgust.

Proximity to moral worth, to maiden purity, to virtuous womanhood, to high Christian character, as infallibly elevate, ennoble, and sanctify, as associations with lawlessness, bestiality, and crime, degrade and ruin and destroy.

If then we desire that emanations should go out from us fairly loaded with influences and powers which are healthful, beautiful, elevating, and benign, we must be clean in person, as well as pure in heart; we must strive to be as faultless in dress as we desire to be engaging in manner; we must bring to our assistance all the aids of taste and art in order to present to the world as far as possible a comely and perfect physique; just as reason and grace are summoned to help us attain a high moral and religious character. In plainer phrase, if your clothes are dirty, wash them, or stay at home; if they are ragged, patch them, or keep out of the street; if you are deformed, employ a tailor or dressmaker of genius; if you have lost a limb, get a Palmer leg; if you have a snaggled tooth, consult Allen of Bond street, for comeliness is a duty as much as health, and so is religion!

